# The Influence of Growth, Maturation and Resistance Training on Muscle-Tendon and Neuromuscular Adaptations: A Narrative Review

**DOI:** 10.3390/sports9050059

**Published:** 2021-05-08

**Authors:** Nakul Tumkur Anil Kumar, Jon L. Oliver, Rhodri S. Lloyd, Jason S. Pedley, John M. Radnor

**Affiliations:** 1 Youth Physical Development Centre, Cardiff School of Sport and Health Sciences, Cardiff Metropolitan University, Cardiff CF23 6XD, UK; joliver@cardiffmet.ac.uk (J.L.O.); rlloyd@cardiffmet.ac.uk (R.S.L.); jpedley@cardiffmet.ac.uk (J.S.P.); jradnor@cardiffmet.ac.uk (J.M.R.); 2 Sport Performance Research Institute New Zealand, Auckland University of Technology, 1010 Auckland, New Zealand; 3 Centre for Sport Science and Human Performance, Waikato Institute of Technology, 3200 Hamilton, New Zealand

**Keywords:** youth, boys, muscle architecture, tendon stiffness, muscle activation, kinetics

## Abstract

The purpose of this article is to provide an overview of the growth, maturation and resistance training-related changes in muscle-tendon and neuromuscular mechanisms in youth, and the subsequent effect on performance. Sprinting, jumping, kicking, and throwing are common movements in sport that have been shown to develop naturally with age, with improvements in performance being attributed to growth and maturity-related changes in neuromuscular mechanisms. These changes include moderate to very large increases in muscle physiological cross-sectional area (CSA), muscle volume and thickness, tendon CSA and stiffness, fascicle length, muscle activation, pre-activation, stretch reflex control accompanied by large reductions in electro-mechanical delay and co-contraction. Furthermore, a limited number of training studies examining neuromuscular changes following four to 20 weeks of resistance training have reported trivial to moderate differences in tendon stiffness, muscle CSA, muscle thickness, and motor unit activation accompanied by reductions in electromechanical delay (EMD) in pre-pubertal children. However, the interaction of maturity- and training-related neuromuscular adaptions remains unclear. An understanding of how different neuromuscular mechanisms adapt in response to growth, maturation and training is important in order to optimise training responsiveness in youth populations. Additionally, the impact that these muscle-tendon and neuromuscular changes have on force producing capabilities underpinning performance is unclear.

## 1. Introduction

Growth and maturation underpin a significant number of natural changes in the neuromuscular system, such as changes in the muscle-tendon architecture and muscle activation, as well as an increase in circulating androgens as youth transition from childhood, through adolescence and into adulthood [1,2,3]. These neuromuscular changes may begin to explain the improvements observed in sprint [4,5,6] and jump performance [4,7,8] as children mature. However, research comparing the magnitude of these maturity-related changes and their implications on the mechanisms driving performance improvements are scarce.

There currently exists a large body of evidence showing the positive influence of resistance training on outcome measures such as jump height [9,10,11,12,13], change in direction speed [14,15], running velocities [10,12] and sprint times [13,14] in youth. However, very few studies have examined the mechanistic changes following resistance training and the subsequent effect on the force producing capabilities that may underpin these training-induced improvements in performance [16,17,18,19,20]. The interaction between growth, maturation and resistance training to promote neuromuscular adaptations in youth is less well understood compared to just growth- and maturity-related changes alone [21,22]. An awareness of how the different muscle-tendon and neuromuscular changes adapt in response to growth, maturation and training is important in order to design more appropriate training programs and optimise training responsiveness in youth populations. Therefore, the aims of this review are to provide an overview of (i) growth and maturity-related changes in muscle-tendon and neuromuscular mechanisms in youth, and (ii) the interaction of the maturity- and training-related changes in muscle-tendon and neuromuscular mechanisms, and their subsequent effect on performance.

## 2. Influence of Growth and Maturation on Muscle-Tendon Structure and Properties

The ability to generate maximal external force at any given velocity is influenced by a series of morphological or structural factors, such as muscle cross-sectional area (CSA), physiological CSA, muscle volume, pennation angle and fascicle length [23]. Physiological CSA differs from anatomical CSA in that the former is a cross-section perpendicular to the muscle fibre direction and is therefore always larger in pennate muscles [24]. While muscle CSA directly correlates with force production [25,26], changes occurring in terms of the specific muscle architecture may also underpin natural strength gains as children transition into adulthood [2]. Furthermore, the role that the tendons have in rapid force production and transmission is also vital for performance and is influenced by its properties [23,27,28]. Prior research has shown that these structural factors undergo growth and maturity-related changes as children transition into adolescence [2,29,30,31].

### 2.1. Muscle Cross-Sectional Area

Cross-sectional area for an individual muscle is the largest CSA along the length of that muscle [32]. Studies have reported increases in muscle CSA during maturation, with some suggesting that the greatest changes occur in boys around the age of 13–15 years [29]. Furthermore, as highlighted in Table 1, adults demonstrate greater muscle thickness than children [33] and older adolescents exhibit greater muscle thickness relative to their younger peers [28,32]. Longitudinal research has shown that adolescents who had experienced their growth spurt increased muscle thickness to a greater extent than those experiencing, or yet to experience peak height velocity (PHV) [34]. Similarly, researchers have observed an approximate threefold difference in quadricep muscle volume and twofold difference in quadricep physiological CSA between boys and men [33].

Muscle CSA, thickness and volume, indicative of muscle hypertrophy [39], have been shown to have a significant influence on absolute force production in adults [40,41,42,43,44]. Cross-sectional studies in youth have also demonstrated a similar relationship, suggesting that maximal voluntary force that can be exerted by a muscle is strongly influenced by size, whatever the age [26,45]. This would imply that increases in muscle size would lead to enhanced force producing capabilities and performance improvements as children mature. This is further supported by recent findings where participants who made large worthwhile changes in jump performance and sprint speed over an 18-month period also experienced large increases in muscle thickness of the vastus lateralis, highlighting the importance of muscle thickness increases underpinning improvements in jump and sprint performance in boys [34]. Cumulatively, these findings highlight that muscle size increases as children mature. This leads to a natural improvement in force production and may explain the improvements in physical performance tests throughout maturation. Practitioners should be aware that these qualities will increase naturally with growth and maturation, and large increases in muscle size with training may need to be observed to have any confidence of training effects above and beyond natural growth and maturation.

### 2.2. Fascicle Length

Within a muscle, fibres are grouped into small bundles termed fascicles [46], and the length of a fascicle is typically measured as the distance between the intersection composed of the superficial aponeurosis and fascicle and the intersection composed of the deep aponeurosis and the fascicle [47]. As shown in Table 1, studies examining differences in fascicle length across maturity groups have reported large to very large differences when comparing boys and men, but small to moderate differences between 14–16-year-old boys [33,35,36]. It was suggested by Kubo et al. [30], that while adults possessed muscle fascicle lengths not significantly longer than that of 15-year-old adolescents, the 15-year olds had significantly longer muscle fascicle lengths than children (see Table 1), possibly implying that fascicle length reaches adult levels at around 15 years of age [2]. In a recent study, Radnor et al. [36] reported that the greatest change in gastrocnemius medialis fascicle length was observed in the group experiencing peak height velocity (PHV; ~10% increase) compared to the group that had already experienced PHV (~9%) or were yet to experience their growth spurt (no change). However, the largest change in vastus lateralis fascicle length occurred in the group that had already gone through PHV (~7%) compared with those experiencing or yet to experience PHV (~5 and ~4%, respectively). The differences in fascicle length being reported vary based on the muscle examined, and the site of observation. These child–adult differences in fascicle length might be caused by the longer limb length of adults compared to children [33,48,49]. Kubo et al. [30] demonstrated that this lengthening of the fascicle with maturation is driven by the muscle catching up with bone growth, which occurs prior to muscle lengthening. The increase in fascicle length might suggest a maturity-related change in fascicle: tendon length ratio, which would have consequences for the contractile properties of the muscle-tendon unit (MTU) as a whole [2,48,50]. However, O’Brien et al. [49] suggested that the increase in the length of the MTU was a result of proportional increase in fascicle, muscle and tendon lengths, implying that the fascicle:tendon length ratio is unlikely to change with maturation.

While studies of animal skeletal muscle suggest that muscle fascicle length plays an important role in determining maximal contraction velocities [51,52], evidence of such a relationship between human skeletal muscle fascicle length and contraction velocity is limited. It has been suggested that longer fascicles may also allow muscles to remain close to optimal length for force production, meaning greater force at longer lengths [53], and allow the muscle to operate effectively over a greater range of motion [54]. With the fascicle:tendon length ratio is unlikely to change during maturation, the longer fascicles in adults or adolescents would allow for greater absolute maximum shortening velocity, while the relative maximum shortening velocity in adults and children would remain equal [48]. With fascicle length strongly influencing the distance over which force is produced [53] and contraction velocities [23], increases in fascicle length would be expected to lead to improved athletic performance [55,56,57]. Radnor et al. [34] reported a small but significant correlation between fascicle length and maximal sprint speed, relative peak force, and relative peak power in boys, thereby suggesting that individuals with longer fascicles can produce quicker movements, but it is an innate quality that may not develop with maturity. While these findings suggest that fascicle length in boys may increase naturally with growth and maturation and could influence force production through greater ranges, it may be beneficial for practitioners to understand the potential innate quality of fascicle length and use this for talent identification purposes [34].

### 2.3. Pennation Angle

Pennation angle of a muscle can be defined as the angle between the muscle’s fascicles and the line of action [51]. Table 1 shows that several studies that have reported changes in pennation angle resulting from maturation to be muscle and site specific [33,36]. Researchers have reported that as an individual transitions from childhood to adulthood, the pennation angle of the vastus lateralis appears to remain fairly consistent [33], while that of the gastrocnemius medialis increases from birth and becomes stable after the adolescent growth spurt [1].

An increase in pennation angle allows for more sarcomeres to be arranged in parallel, meaning more contractile tissue is able to attach to a given area of aponeurosis or tendon resulting in greater PCSA [58,59], which in turn facilitates greater force production by the muscle [59]. Fascicle pennation not only influences strength by enabling a greater PCSA, but it is functionally important due to the process known as “*gearing*” [60]. Due to the pennation angle, and the fact that fibres rotate as they shorten, during muscle contractions the muscle fascicles may shorten at a rate different from the whole muscle, and the ratio of these velocities is its gearing [60]. Therefore, fascicles are not required to shorten as much as the whole muscle, resulting in the muscle operating on a more optimal region of its force-velocity curve, and working at a favourable region of its length-tension relationship over a longer period. This maximises the force that the muscle can develop, without impacting on the capacity for rapid movement production [61].

The pennation angle of the lateral gastrocnemius has been shown to correlate with higher early rate of force development (RFD) in adults during drop jumps [62]. Researchers have speculated that this could be a result of the indirect line of pull of fibres in pennate muscles, resulting in the muscle having an increased ability to resist external forces, greater muscular stiffness and isometric-like qualities during muscle lengthening [63]. However, previous studies have reported that smaller pennation angles of the VL are associated with greater sprinting ability in boys [34], and this could be due to the fact that smaller pennation angles would allow for longer fascicles [63]. It is useful for practitioners to understand how maturity-related changes in pennation angle are site-specific, and that the requirement for either large or small pennation angles could be dependent on the task, specific muscle, and population.

Studies that have examined growth- and maturity-related changes in muscle structure and morphological factors are summarised in Table 1. The table also highlights the magnitude of differences between children, adolescents and adults. Effect sizes observed for muscle size appear greater than those for muscle architecture, with very large differences being observed for muscle PCSA as well as muscle volume and thickness. Studies have reported significant correlations between muscle thickness and pennation angle [64,65], suggesting that an increase in muscle thickness is accompanied by changes in pennation angle [64]. The effect sizes for changes in muscle architecture appear to be more site-dependent, with moderate differences being observed for VL fascicle length and small differences for GM fascicle length, when comparing pre- and post-pubertal boys.

### 2.4. Tendon Architecture and Stiffness

Tendons are interposed between muscles and bones to form an MTU which transmits muscular forces directly to the bone, thereby creating movement and stability about a joint [66,67]. Tendon stiffness can be referred to as its resistance to elongation when a force is applied, and is attributed to the greater number of spring-like materials arranged in parallel [2]. The dimensions of the tendons largely affect their function and properties, and while longer, more compliant tendons are suggested to more readily absorb energy [68], they have been linked to longer electromechanical delay (EMD; a delay in the detection of force onset) [2,28,69]. Shorter and thicker tendons (greater CSA) are stiffer and more effective at transferring muscular forces to bone and thereby associated with greater RFD and reduced EMD [2,62].

The level of tendon stiffness or compliance can influence maximal muscular force [23]. An increase in tendon stiffness can be explained by increases in tendon size or Young’s modulus [70,71]. Young’s modulus, which can be defined as the ratio of tensile stress to tensile strain, is an inherent property of any viscoelastic structure to withstand changes in length under tension and compression [72]. Young’s modulus can be affected by tendon microstructural changes such as collagen fibril diameter [73], increased collagen cross-linking [74], and reduced collagen crimping [75]. Table 2 highlights prior research that has examined changes in tendon dimensions across maturity groups and reported that plantar flexor tendon length was 20% greater in adults than in younger boys (~11 years), with no significant difference being observed between adults and older boys (~14 years) [31]. This may suggest that these variables become stable in boys around the age of 14 years, which is the average time of PHV [76]. Prior studies have also reported an approximate two-fold increase in patellar tendon CSA from childhood to adulthood [77], suggesting an increased tendon stiffness given the association with tendon CSA [2,62]. Increases approximately two-fold in magnitude have been reported for stiffness of both the patellar [77,78] and Achilles (adults 2.5 times greater than children) [79] tendons with age. These studies also reported little to no significant difference between older children (~14 years) and adults, further supporting the suggestion that adult values may be reached approximately at the time of PHV [30,31,77]. With increases in tendon stiffness being suggested to inversely affect EMD [28,80,81,82], and elicit an improved stretch reflex amplitude [2], the growth and maturity-related changes may lead to an increased RFD and greater force production. In a study examining the implications of differences in dynamic muscle-tendon behaviour, Waugh et al. [83] reported that during hopping, MTU length change in children was accomplished with greater muscle excursion in children compared to adults, suggesting greater energy cost of producing mechanical work. The authors suggested that although both adults and children choose movement frequencies that maximise elastic energy storage potential of the tendon, children’s energy saving mechanisms might not be as effective as adults, and this was attributed to differential development of muscle and tendon mechanics during childhood [83]. The findings indicate that as children mature, they develop greater tendon stiffness that might positively influence the energy-saving mechanisms. Practitioners need to be aware of the impact this could have on performance and ensure the prescription of appropriate training to allow for the development of these qualities alongside the natural development from growth and maturation.

A summary of studies examining the magnitude of differences in tendon architecture between adults and children as a result of growth and maturation is provided in Table 2. Extremely large differences between children and adults in tendon CSA are accompanied by extremely large differences in tendon stiffness. Differences in tendon length appear to be greater for the patellar tendon compared to Achilles tendon, and this could potentially be explained by greater growth occurring in the femur compared to tibia during childhood and adolescence [84].

## 3. Influence of Growth and Maturation on Neural Mechanisms

Research has consistently indicated that children differ from adults in several muscular performance attributes, such as maximal force production and RFD [69,85,86]. These attributes are closely associated with performance of activities such as jumping and sprinting [2,34,87,88]. In addition to the growth and maturity-related development of muscle-tendon structure and properties, the ability to generate high levels of muscle activity develops with maturity and will influence the ability to generate force rapidly [69]. There are a number of neural mechanisms that improve with maturation that could partly account for the differences in these qualities, such as variance in muscle activation rates, differential motor unit recruitment, reduced electromechanical delay, increased muscle pre-activation, reduced agonist–antagonist co-contraction, and improved stretch reflex control and conduction velocity [69,89].

### 3.1. Muscle Activation

When considering the production of force, muscular activation plays a vital role [90]. While maximal muscle activation refers to all available motor units being recruited and driven to their maximal firing rates [90], voluntary activation is commonly defined as the level of neural drive to muscles during a maximal voluntary contraction (MVC), and a lack of full voluntary activation is termed as voluntary activation deficit [91,92]. As shown in Table 3, studies have reported that activation deficit during an MVC decreases significantly from pre- to post-puberty, with 7 year old children displaying deficit levels approximately three times greater than 10 year olds, four times greater than 11 year olds and nine times greater than adults [93,94]. The increase in levels of voluntary activation with age is suggested to reflect changes in central command, where muscle control may involve enhanced motor unit recruitment, an increased firing rate of activated motor units, change in motor unit firing pattern, or an increase in conduction velocity within motor pathways [93,94].

The size principle for the orderly recruitment of motor units ensures that the slowest, most fatigue-resistant motor units are recruited first for any task, with the faster motor units with greater force-producing capabilities being reserved for high intensity tasks where they can provide high forces for a short period of time [95,96,97]. However, research has indicated that the recruitment order of the motor units differs based on the velocity of the contraction [98], suggesting that the sequence of activation could be modified or even reversed in rapid voluntary movements [99,100]. However, it is unclear as to which motor units are less activated in youth, and whether the lower activation levels are also a result of potentially inferior motor unit synchronisation.

Given that type-II motor units have a faster contraction velocity than type-I [69], differential motor unit recruitment should have implications that extend beyond just maximal force [69]. Several studies have demonstrated that RFD during maximal isometric contractions is higher in adults compared to children (rate of torque development in men is approximately 4.5 times greater than boys), and this difference is still observed when normalised to muscle cross-sectional area [69,89,101]. It can be speculated that the differences in RFD could link back to differences in motor unit recruitment [69], evidenced by an association between type-II motor units and peak RFD, especially in the early phase of muscle contraction [102,103]. The lower RFD levels in children are thereby suggested to be a reflection of lesser utilisation of type-II motor units compared to adults [69].

During fast maximal muscle contractions, lesser activation of type-II motor units is suggested to result in higher levels of EMD [69]. This delay has been reported to be approximately 50% longer in boys and girls compared to adults [81,89], suggesting lesser activation of the type-II motor units in children [69,101]. Additionally, although an inverse relationship has been suggested between tendon stiffness and EMD [2], certain studies have reported that the MTU stiffness only accounts for <20% of variance in EMD changes [28,81,82]. These findings suggest that lower muscle activation, as well as lesser recruitment and utilisation of higher-threshold motor units in children could also account for child–adult differences in EMD [80,81,82,93].

Mean power frequency of an electromyography (EMG) signal, which is the mean relative distribution of EMG frequencies, has previously been used to infer differential motor unit recruitment, with men being reported to have values 20% greater than boys [82]. The authors attributed this difference to the greater utilisation of type-II motor units in adults [82], and this was further supported by the observation of a greater drop off in mean power frequency in men (~50%) than in boys (~12%), following a fatiguing isometric MVC protocol. Decreases in mean power frequency, during intense fatiguing contractions, have been reported to be greater in individuals with higher composition and utilisation of type-II motor units [104]. It has also been hypothesised that the difference in mean power drop off could be due to greater lactic acid accumulation in men compared to boys, an occurrence that is expected more of type-II than of type-I motor units [69,104].

The findings of the studies highlighted in Table 3 suggest that as children mature, they are better able to recruit higher threshold motor units. This improved differential motor unit recruitment is accompanied by moderate to very large increases in muscle activation which could result in growth related improvements in maximal force producing capabilities and an enhanced ability to rapidly produce force, potentially leading to increases in RFD, peak force and impulse. Given the magnitude of changes in muscle activation strategies that youths experience as they mature, and with prior research suggesting that training-related changes in pre-pubertal children are primarily neural [105], practitioners should be aware that they might benefit from designing training programs that are complementary to the natural adaptive processes.

### 3.2. Muscle Pre-Activation

Pre-activation is a term that is commonly used to refer to the levels of muscle activity prior to an impact or landing, and which is prominent in the early phase of stretch shortening cycle (SSC) sequence [109,110]. Pre-activation plays an important role in regulating ankle stiffness during rebounding and jumping activities [111,112], and is vital for torque enhancement in the knee extensors and plantar flexors during such activities [109,113].

During activities such as hopping, children have been reported to have significantly lower pre-activation compared to adults, particularly at higher movement speeds [106,111,112,114]. It has been suggested that the delayed and lower levels of pre-activation could be explained by a relationship between maturation and the ability to predict an event; the behaviour of children prior to landing has at times been compared to that of ‘untrained jumpers’ [106,115,116]. This delayed and lower level of pre-activation results in longer ground contact times, which in turn reduces the magnitude of elastic energy contribution leading to sub-optimal SSC function and thereby a lower peak force and impulse in the subsequent concentric phase [2,107,117]. Additionally, lower levels of pre-activation have been suggested to cause the peak vertical ground reaction force to manifest as an impact peak (defined as a force of high magnitude resulting from the collision of two bodies over a relatively short period), during the early phase of ground contact [118,119]. Prior research has reported that a greater proportion of pre-PHV boys, compared to post-PHV, display the presence of an impact peak [88]. This reduction in prevalence of an impact peak in older children can be attributed to pre-activation improving with age, as evidenced by significantly greater muscle pre-activity being observed in 15-year old boys compared to nine- and 12-year old boys [107], and greater background muscle activity compared to men [114].

Improved SSC function associated with the maturity-related increases in the levels of pre-activation allows for greater joint stiffness during the braking phase of the SSC and enables more rapid force production upon ground contact [2,120]. This may result in greater RFD immediately following ground contact, a shorter ground contact time and reduced centre of mass displacement [121]. The development of these feed-forward mechanisms with growth and maturation may allow for children to become more pre-active than reactive, which might be useful for practitioners to consider when implementing or progressing training tasks such as plyometrics. Additionally, improvements in pre-activation could also play a role in reducing risk of non-contact injuries.

### 3.3. Stretch Reflex Control

When examining muscle activity during landings or impact, mean EMG values between 30 and 60 ms, 61 and 90 ms and 91 and 120 ms can be used to represent short-, medium- and long-latency stretch reflex components, respectively [111]. While the short-latency stretch reflex signifies muscle activity as a result of spinal involuntary commands, the medium- and long-latency stretch reflexes signify activation resulting from supraspinal commands [114,122].

Research has utilised plyometric movements to study stretch reflex activity, based on the fact that the reflex amplitude influences MTU stiffness which in turn affects SSC performance [106,114]. When quantifying the stretch reflex by means of plyometric exercises, higher amplitudes of stretch reflex have been observed in adults compared to children, with children exhibiting a greater reliance on longer-latency stretch reflexes [106,107,114], thereby resulting in sub-optimal MTU stiffness [106]. However, children have been shown to regulate lower-limb stiffness more effectively as they mature, and this has been attributed to a greater utilisation of short-latency stretch reflexes [114] which may underpin the increases in spring like behaviour displayed by more mature youth [88]. The improved utilisation of these stretch reflexes has been attributed in part to improved spindle sensitivity and maturation of the sensorimotor pathways [114,123]. Additionally, increases in muscle pre-activation have also been suggested to facilitate a greater short-latency stretch reflex response [124]. The amplitude and timing of the stretch reflex has been evidenced to underpin lower limb stiffness [111,112,125], and increased stiffness leads to shorter ground contact times and in turn a more efficient reutilisation of elastic energy due to a quick transition between eccentric and concentric phases [117,126]. Cumulatively, there exists sufficient evidence to suggest that the stretch reflex contributes significantly to rapid force generation during touchdown in activities such as jumping, hopping and running [127]. Practitioners need to be aware that although maturity-related improvements in the feed-back mechanisms positively influence lower limb stiffness and hence force output during SSC-driven activities, the amplitude of the short-latency stretch reflex might vary considerably depending on the activity.

### 3.4. Co-Contraction

Co-contraction refers to the simultaneous contraction of the agonist and antagonist muscles about a joint, and is known to stabilise limb movements [23,128]. While this co-contraction may increase joint stability, high levels of antagonist activity result in an increase in agonistic muscle energy expenditure to complete a task [129]. During activities such as jumping and running, when the magnitude of co-contraction exposes the MTU to excessive tensile forces, the activity of the Golgi tendon organs increases and results in an inhibition of the motoneurons innervating the agonist muscle, and facilitation of those innervating the antagonist muscles [130]. This then may lead to increased ground contact times and lower force outputs due to a reduction in the efficiency of the SSC [2]. 

As seen in Table 3, research has suggested that co-contraction decreases with growth and maturation, i.e., the co-contraction index has been reported to be almost twice as high in 10–12-year-olds compared to 15–16-year-olds [85,93,108]. This maturity-related reduction in co-contraction is underpinned by a greater density and size of the Golgi tendon organs in children compared to adults [131]. During maturation, the Golgi tendon organs undergo a process of desensitisation leading to a reduction in the magnitude of co-contraction, which results in decreased agonist inhibition thereby allowing for a more efficiently functioning SSC and an increased net force [2]. Such maturity-related decreases in co-contraction allow children to naturally become more efficient and may subsequently have a positive effect on performance during SSC driven activities.

Studies that have examined growth- and maturity related changes in the neural mechanisms are summarised in Table 3. The magnitude of differences between adults and children in neural mechanisms appear to be similar to those observed for structural factors, with very large differences in muscle activation and co-contraction, large differences in EMD, and moderate differences in mean power frequency. When comparing pre- and post-pubertal boys, large differences were observed in neural mechanisms such as short latency stretch-reflex activity, with the magnitude being similar to differences observed in muscle size but greater than those observed for muscle architecture.

## 4. Effect of Resistance Training on Muscle-Tendon and Neuromuscular Systems

Resistance training involves the progressive use of a wide range of resistive loads and a variety of training modalities to increase an individual’s ability to exert or resist force [132,133,134]. Traditional resistance training involves exercises such as squatting, pressing and pulling where a significant amount of the movement duration, towards the end of the range of motion, involves a deceleration phase [135,136,137]. While this method is vital for developing strength [138,139], there is the need for movements which are more mechanically specific to performance, such as ballistic exercises, plyometrics, and weightlifting exercises [140]. Prior research has defined ballistic contractions as those in which there is no opportunity to alter a movement pattern once it is executed and attributed this to the short duration of the movement [99,141]. Ballistic exercises of a dynamic nature are typically utilised as a method of training to improve maximal power output [142]. Plyometric exercises, characterised by rapid SSC actions, are also utilised within power training programmes and are typically performed with little or no external resistance [140]. While weightlifting movements, such as the clean and jerk and the snatch, are similar to ballistic exercises [140], the two differ in that weightlifting involves a specific set of movements (typically involving a concentric phase only or a concentric phase followed by an eccentric phase) and might often be performed with a higher resistance and therefore lower velocities compared to ballistic exercises [137,140,143]. There exists a substantial amount of empirical evidence indicating that resistance training is safe for children and adolescents [144,145]. Injury epidemiology studies have shown that injuries resulting from resistance training are far less common than those associated with popular sports such as American football, gymnastics, basketball, rugby, or soccer [145,146,147]. Studies have reported positive correlations between motor skill performance and the intensity (% 1 repetition maximum) of the resistance training program [148], suggesting that children and adolescents can make improvements in performance following exercise at a high intensity. Meta-analytical data including 42 studies showed that the average resistance training prescription for youth was typically two to three sets, with 8–15 repetitions, using loads of 60–80% 1 repetition maximum, with training periods lasting approximately 10 weeks [149]. However, a more recent meta-analysis that examined resistance training specifically in young athletes attempted to examine the optimal dose–response for youths. The research showed that the most effective training prescription for strength gains required longer periods of training (>23 weeks), the use of heavier loads (80–90% of 1 repetition maximum) and greater training volumes (5 sets of 6-8 repetitions) [150]. Cumulatively, it would appear that as a child becomes more experienced and acquires higher levels of athleticism, resistance training prescription would need to change, especially in terms of the volume and intensity of training. Additionally, the development of physical literacy is deemed of equal importance, since physically literate youths perform exercises with greater technical ability, confidence and competence [151]. Researchers have suggested that a combination of supervised, structured training along with free play can maximize children’s ability as well as their confidence and adherence to physical activity long term [151,152,153]. Owing to its numerous health benefits, numerous professional organisations promote resistance training as a safe, worthwhile and necessary activity for youth to engage in [133,154,155].

While the effect of resistance training on measures of jump height [9,10,11,12,13] and running velocities [10,12] in youth have been thoroughly examined, studies investigating muscle-tendon and neuromuscular adaptations following resistance training are sparse [16,17,18,19,20,156,157]. Although limited in number, Table 4 highlights studies that have examined resistance training-related structural and morphological changes in youth. While the studies reported increases in maximal strength and jump height following resistance training, in terms of muscle morphology they observed moderate to large changes in adolescents and trivial to small changes in pre-pubertal children [20,157,158,159]. In accordance with prior research, the authors attributed the training-induced gains in pre-pubertal children primarily to neural adaptations [105,160,161].

While resistance training-related structural changes in pre-pubertal children are suggested to be minimal [105,160] there are several studies that have observed morphological changes in children across maturity groups following resistance training [20,156,158]. While the study by Granacher et al. [159] did not elicit significant hypertrophic changes in pre-pubertal participants, it could be argued that because the study was examining the effect of strength training, the prescribed rest periods between sets (3–4 min) were not rest periods that would be prescribed when the goal of the program is to elicit hypertrophic adaptations [162]. Conversely, while McKinlay et al. [156] utilised similar repetitions, sets and intensities as in Granacher et al. [159], their rest periods were restricted to 60–90 s and the authors reported significant baseline to post-intervention increases in muscle thickness in boys aged 12–13 years. It is interesting to note that while both studies reported an increase in knee extensor peak torque [156,159], only the study by McKinlay et al. [156] reported an increase in countermovement jump height. This increase could potentially be explained by muscle thickness being suggested to positively influence jump performance [34]. Additionally, prior research has suggested that when trying to elicit meaningful changes, longer exposures to training (>23 sessions) with appropriate training stimuli are required to elicit significant adaptations [163]. It is important to note that the participants in Granacher et al. [159] received 20 training sessions, while those in McKinlay et al. [156] received 24 sessions. This suggests that the distinct neuromuscular adaptations observed following resistance training in children might be specific to the training program design, duration of the program and maturity status of the participants [48,164,165].

In adults, increased pennation angle has been reported following heavy strength training [65,166], and this results in a greater number of sarcomeres arranged in parallel within a given CSA, which is associated with increased maximal force producing capabilities [167,168,169]. Research suggests that fascicle length in adults increases following resistance training with light loads [170] as well as jump and sprint training [171], indicative of adaptations potentially being associated with the force-velocity characteristics of the exercises. However, increases in fascicle length have also been observed following heavy eccentric training (accentuated and eccentric-only) in adults [53,54,172], with the adaptation being suggested to be a protective mechanism against exercise-induced muscle damage in a subsequent eccentric exercise session [173]. To the author’s knowledge, research is yet to examine the effects of resistance training on muscle pennation angle and fascicle length in youth.

When comparing a control group to an experimental group, Waugh et al. [157] reported resistance training to elicit significant increases in tendon stiffness with no significant change in tendon CSA. This indicates that although ~78% of child–adult differences in tendon stiffness can be attributed to loading due to increased body mass and force production capabilities associated with maturation, increased external loading due to resistance training also promotes improvements in tendon stiffness. Studies have reported an increase in tendon stiffness in children following resistance training to be accompanied by a paralleled decrease in EMD [19,80,93,157]. Given this association of tendon stiffness with a reduction in EMD [28,81] and improved rate and efficiency of transfer of muscular forces [27] and amplitude of stretch reflex [2], such increases in tendon stiffness following resistance training might allow for greater RFD [2,85]. This suggestion can potentially be supported by the findings of several studies which have reported resistance training-related increases in eccentric and concentric RFD following resistance training in adults [174,175,176].

Very few studies have directly examined the effect of training on maximal voluntary activation in children. Ramsay et al. [20] observed significant improvements in strength measures resulting from resistance training and while they found no significant differences in muscle CSA, the authors reported a trend towards an increased percentage of motor unit activation in the experimental group. Researchers have speculated that children might be able to make larger resistance training-related increases in voluntary activation compared to adults, and this has been attributed to children’s comparatively lower levels of voluntary activation [69,93,177], suggesting a larger potential for adaptive change. With an increase in motor unit activation being linked to augmentation in force production, such increases following resistance training may result in an enhanced ability to produce force.

To the author’s knowledge, no previous studies have directly investigated the effect of resistance training on differential motor unit recruitment; however, an increased RFD in response to explosive sport training has been reported in young gymnasts and this has been linked to increased type-II motor unit recruitment and higher motor unit synchronisation [17,69]. Such training-related improvements in the ability to recruit higher threshold motor units would result in a reduction in EMD and an enhanced ability to rapidly produce high levels of force [2], potentially reflected in the improved jump [9,10,11,12,13] and sprint times [13,14] observed in children following resistance training. While other factors such as pre-activation, reflex control, co-contraction and activation deficit also affect the force producing capabilities that underpin performance, their responses to resistance training are yet to be investigated. Table 4 summarises studies that have examined neuromuscular adaptations following resistance training in youth and highlights the magnitude of change from baseline to post-intervention.

Further longitudinal research is required to determine how children across different maturity groups respond to resistance training and determine how the structural and neural responses may differ dependent on type of training and the interaction with growth and maturation. Future research should also investigate the effect of long-term training interventions on neural mechanisms such as differential motor unit recruitment, pre-activation, reflex control, co-contraction and activation deficit in youth, and the subsequent effect on performance of sprinting, jumping and rebound activities.

## 5. Conclusions

The current review aimed to provide an overview of existing research that has examined muscle-tendon and neuromuscular changes associated with growth, maturation and training and how this influences force production. Studies have reported growth and maturation to elicit moderate to very large changes in muscle physiological CSA, volume and thickness, tendon CSA and tendon stiffness, fascicle length, muscle activation, pre-activation and stretch reflex control accompanied by large reductions in EMD and co-contraction. Although research examining the changes in neuromuscular mechanisms following resistance training in children across maturity groups is scarce, the available literature reports trivial to moderate differences in tendon stiffness, muscle CSA and thickness, as well as small increases in motor unit activation and small reductions in EMD in pre-pubertal children.

## Figures and Tables

**Table 1:** Effects of growth and maturation on muscle morphology in children (for multiple groups difference and effect size are expressed for consecutive pairs).

Author	Developmental Change	Sample Age Range	Findings		
			Values	Difference (%)	Effect Size (g)
O’ Brien et al. [33]	Muscle PCSA, Volume, Pennation Angle and Fascicle Length	10 men aged 28.2 ± 3.6 years and 10 boys aged 8.9 ± 0.7 years not participating in organised sport of physical activity outside school.	Muscle PCSA—Men vs. Boys (cm^2^)		
			(VL) 74.04 ± 17.04 vs. 31.43 ± 7.40	136%	3.24
			(VM) 55.40 ± 16.12 vs. 21.71 ± 5.40	155%	2.80
			(VI) 59.28 ± 17.87 vs. 30.99 ± 6.70	91%	2.10
			(RF) 43.06 ± 11.88 vs. 20.46 ± 4.80	110%	2.49
			Muscle Volume—Men vs. Boys (cm^3^)		
			(VL) 691.22 ± 147.90 vs. 236.13 ± 42.30	193%	4.18
			(VM) 523.18 ± 133.80 vs. 155.46 ± 29.90	237%	3.79
			(VI) 557.58 ± 143.10 vs. 200.81 ± 47.60	178%	3.35
			(RF) 280.71 ± 66.10 vs. 116.17 ± 23.90	142%	3.31
			Pennation Angle—Men vs. Boys (deg)		
			(VL) 15.4 ± 4.3 vs. 15.9 ± 2.3	4%	0.16
			(VM) 25.4 ± 7.6 vs. 23.3 ± 4.8	9%	0.33
			(VI) 13.6 ± 3.4 vs. 11.8 ± 1.6	15%	0.65
			(RF) 29.4 ± 10.2 vs. 20.8 ± 4.4	41%	1.10
			Fascicle Length—Men vs. Boys (mm)		
			(VL) 94.5 ± 15.4 vs. 76.6 ± 10.6	23%	1.35
			(VM) 95.9 ± 15.5 vs. 72.7 ± 7.9	32%	1.89
			(VI) 95.3 ± 11.2 vs. 64.7 ± 6.8	47%	3.30
			(RF) 67.7 ± 16.5 vs. 58.4 ± 15.1	16%	0.59
Kubo et al. [35]	Muscle Thickness and Fascicle Length	23 sedentary/moderately active men aged 22.2 ± 2.2 years and 20 boys aged 11.2 ± 1.1 years not participating in organised sport of physical activity outside school.	Muscle thickness—Men vs. Boys (mm)		
			(KE) 24.1 ± 3.3 vs. 17.5 ± 2.1	38%	2.35
			(PF) 21.3 ± 2.7 vs. 14.4 ± 1.4	48%	3.14
			Fascicle length—Men vs. Boys (mm)		
			(KE) 90.2 ± 7.9 vs. 65.7 ± 4.1	37%	3.81
			(PF) 56.2 ± 6.2 vs. 47.2 ± 6.2	19%	1.45
Radnor et al. [36]	Muscle Thickness, Pennation Angle and Fascicle Length	57 boys aged 12.45 ± 0.54 years (G1), 32 boys aged 14.06 ± 0.68 years (G2), and 37 boys aged 15.81 ± 0.97 years (G3). All boys were involved in regular sport and P.E programs.	Muscle Thickness—G1 vs. G2 vs. G3 (mm)		
			(GM) 14.7 ± 1.6 vs. 16.8 ± 2.4 vs. 18.1 ± 3.1	14%, 8%	1.09, 0.46
			(VL) 18.3 ± 2.2 vs. 21.3 ± 2.8 vs. 23.8 ± 3.7	16%, 12%	0.92, 0.75
			Pennation Angle—G1 vs. G2 vs. G3 (deg)		
			(GM) 19.25 ± 3.07 vs. 20.52 ± 3.60 vs. 22.83 ± 3.87	7%, 11%	0.39, 0.62
			(VL) 16.48 ± 3.22 vs. 17.53 ± 3.98 vs. 18.36 ± 2.74	6%, 5%	0.30, 0.25
			Fascicle Length—G1 vs. G2 vs. G3 (mm)		
			(GM) 45.5 ± 8.0 vs. 49.1 ± 9.4 vs. 47.5 ± 9.8	8%, 3%	0.42, 0.17
			(VL) 66.4 ± 13.2 vs. 73.4 ± 15.6 vs. 77.5 ± 19.8	11%, 6%	0.50, 0.23
Cunha et al. [37]	Muscle CSA, Muscle Thickness, Muscle Volume, Pennation angle and Fascicle Length	15 boys aged 14.5 ± 0.8 years (G1) and 19 boys aged 16.6 ± 1.2 years (G2). All boys were engaged in formal football training.	Muscle CSA—G1 vs. G2 (cm^2^)		
			(RF) 9.8 ± 1.9 vs. 10.3 ± 2.0	5%	0.26
			Muscle Thickness—G1 vs. G2 (cm)		
			(KE) 3.6 ± 0.6 vs. 3.8 ± 0.6	6%	0.33
			Muscle Volume—G1 vs. G2 (mL)		
			(KE) 1526 ± 307 vs. 1814 ± 410	19%	0.78
			Muscle Pennation Angle—G1 vs. G2 (deg)		
			(VL) 15.0 ± 2.3 vs. 14.3 ± 3.2	5%	0.25
			Muscle Fascicle Length—G1 vs. G2 (cm)		
			(VL) 8.3 ± 1.4 vs. 8.9 ± 1.6	7%	0.40

Effect size (g): <0.2 (trivial), 0.20–0.59 (small), 0.60–1.19 (moderate), 1.20–1.99 (large), 2.00–3.99 (very large), and >4.0 (extremely large) [38]. (GM—gastrocnemius medialis, KE—knee extensors, PCSA—physiological cross-sectional area, PF—plantar flexors, RF—rectus femoris, VL—vastus lateralis, VM—vastus medialis and VI—vastus intermedius).

**Table 2:** Effects of growth and maturation on tendon architecture and properties in children (for multiple groups difference and effect size are expressed for consecutive pairs).

Author	Developmental Change	Sample Age Range	Findings		
			Values	Difference (%)	Effect Size (g)
O’Brien et al. [78]	Tendon CSA and Tendon stiffness	10 sedentary men aged 28.2 ± 3.6 years and 10 boys aged 8.9 ± 0.7 years	Patellar Tendon CSA—Men vs. Boys (mm^2^)		
			114.8 ± 17.8 vs. 75.3 ± 15.0	52%	2.40
			Patellar Tendon stiffness—Men vs. Boys (N/mm)		
			1076 ± 87 vs. 555 ± 71	94%	6.56
Kubo et al. [77]	Tendon CSA, Tendon Length and Tendon Stiffness	22 adults aged 22.3 ± 0.4 years, 21 children aged 11.2 ± 0.2 years (G1) and 18 children aged 13.8 ± 0.1 years (G2)	Patellar Tendon CSA—Adults vs. G2 vs. G1 (mm^2^)		
			82.7 ± 2.1 vs. 65.4 ± 2.8 vs. 49.2 ± 2.3	26%, 33%	7.10, 6.37
			Patellar Tendon length—Adults vs. G2 vs. G1 (mm)		
			47.0 ± 0.8 vs. 45.3 ± 0.6 vs. 38.5 ± 0.8	4%, 18%	2.37, 9.51
			Patellar Tendon stiffness—Adults vs. G2 vs. G1 (N/mm)		
			1507.2 ± 148.1 vs. 1211.9 ± 136.0 vs. 742.9 ± 55.2	24%, 63%	2.07, 4.66
Waugh et al. [79]	Tendon Stiffness	10 men aged 27 ± 2.0 years and nine women aged 24.8 ± 3.2 years (Adults). 21 children aged 6.4 ± 0.8 years (G1), and 29 children aged 9.1 ± 0.5 years (G2)	Achilles Tendon Stiffness—Adults vs. G2 vs. G1 (N/mm)		
			259.2 ± 44.2 vs. 162.4 ± 42.9 vs. 100.8 ± 30.4	60%, 61%	2.23, 1.61
Kubo et al. [31]	Tendon CSA and Tendon Length	23 men aged 22.2 ± 2.2 years, 22 children aged 11.2 ± 1.1 years (G1) and 19 children aged 13.8 ± 0.6 years (G2)	Achilles Tendon CSA—Adults vs. G2 vs. G1 (mm^2^)		
			74.7 ± 14.7 vs. 76.9 ± 16.7 vs. 60.1 ± 13.6	3%, 28%	0.14, 1.11
			Achilles Tendon Length—Adults vs. G2 vs. G1 (mm)		
			275.1 ± 20.8 vs. 263.9 ± 17.5 vs. 229.1 ± 15.2	4%, 15%	0.58, 2.13
O’Brien et al. [49]	Tendon Length	10 sedentary men aged 28.2 ± 3.6 years and nine boys aged 8.9 ± 0.7 years who did not participate in any organised sport or physical activity outside school	Tendon length—Men vs. Boys (mm)		
			(VL) 51.7 ± 3.4 vs. 42.2 ± 3	23%	2.95
			(VM) 63 ± 4.8 vs. 49 ± 5.3	29%	2.78
			(VI) 30.2 ± 3.2 vs. 25 ± 3.9	21%	1.47
			(RF) 124.1 ± 7.7 vs. 96.9 ± 3.8	28%	4.40
Kubo et al. [35]	Tendon Length, Tendon Thickness and Tendon Stiffness	23 sedentary men aged 22.2 ± 2.2 years and 20 boys aged 11.2 ± 1.1 years not involved in any specific training program	Tendon length—Men vs. Boys (mm)		
			(KE) 313.8 ± 15.6 vs. 269 ± 15.3	17%	2.90
			(PF) 275.1 ± 20.8 vs. 229.1 ± 15.2	20%	2.50
			Tendon thickness Men vs. Boys (mm)		
			(KE) 3.30 ± 0.38 vs. 2.61 ± 0.30	26%	2.00
			(PF) 5.14 ± 0.17 vs. 4.72 ± 0.46	9%	1.25
			Tendon stiffness—Men vs. Boys (N/mm)		
			(KE) 57.6 ± 19.8 vs. 23.2 ± 14.0	148%	1.98
			(PF) 35.3 ± 13.1 vs. 20.3 ± 9.5	74%	1.30

Effect size (g): <0.2 (trivial), 0.20–0.59 (small), 0.60–1.19 (moderate), 1.20–1.99 (large), 2.00–3.99 (very large), and >4.0 (extremely large) [38]. (CSA—cross-sectional area, KE—knee extensors, PF—plantar flexors, RF—rectus femoris, VL—vastus lateralis, VM—vastus medialis and VI—vastus intermedius).

**Table 3:** Effects of growth and maturation on neural mechanisms in children (for multiple groups difference and effect size are expressed for consecutive pairs).

Author	Developmental Change	Sample Age Range	Test	Findings		
				Values	Difference (%)	Effect Size (g)
Grosset et al. [93]	Muscle Activation	9 sedentary adults aged 21 ± 2.3 years, 6 children aged 7 years (G7), 7 children aged 8 years (G8), 8 children aged 9 years (G9), 11 children aged 10 years (G10) and 5 children aged 11 years (G11)	MVC isometric plantar flexion	TS Amplitude—G7 vs. G8 vs. G9 vs. G10 vs. G11 vs. Adults (µV)		
				189 ± 38 vs. 216 ± 45 vs. 286 ± 81 vs. 289 ± 92 vs. 365 ± 109 vs. 641 ± 122	14%, 32%, 1%, 26%, 76%	0.64, 1.05, 0.03, 0.78, 2.34
Halin et al. [82]	Differential Motor Unit Recruitment	12 men aged 21.5 ± 4.5 years and 15 young boys aged 10.5 ± 0.9 years, all physically active but not involved in intensive training	MVC isometric elbow flexion	Bicep Brachii MPF—Men vs. Boys (Hz)		
				106.78 ± 30.88 vs. 86.77 ± 14.02	23%	0.87
Falk et al. [81]	Electromechanical Delay	16 men aged 22.1 ± 2.8 years and 15 boys aged 9.6 ± 1.6 years, all physically active	MVC isometric elbow flexion and extension	Bicep Brachii EMD (flexion)—Men vs. Boys (ms)		
				47.6 ± 17.5 vs. 75.5 ± 28.4	59%	1.17
				Tricep Brachii EMD (extension)—Men vs. Boys (ms)		
				38 ± 12 vs. 65 ± 15 Ŧ	71%	1.98
Lazaridis et al. [106]	Pre-activation	12 adult males aged 25 ± 2.7 years 12 and prepubescent boys aged 9.8 ± 0.6 years, all untrained	20 cm drop jump	Preactivation EMG Duration—Men vs. Boys (ms)		
				(GM) 58 ± 19 vs. 35 ± 17 Ŧ	66%	1.28
				(SOL) 47 ± 18 vs. 28 ± 7 Ŧ	68%	1.39
				(TA) 41 ± 17 vs. 29 ± 12 Ŧ	41%	0.82
				Preactivation Amplitude—Men vs. Boys (normalised to max)		
				(GM) 0.2 ± 0.8 vs. 0.1 ± 0.7 Ŧ	29%	0.05
				(SOL) 0.1 ± 0.7 vs. 0.1 ± 0.6 Ŧ	27%	0.05
				(TA) 0.3 ± 0.2 vs. 0.1 ± 0.1 Ŧ	79%	0.78
Lloyd et al. [107]	Stretch reflex activity	11 boys aged 9.44 ± 0.27 (G9), 11 boys aged 12.68 ± 0.30 (G12), and 10 boys aged 15.89 ± 0.31 (G15), physically active but not involved in any strength and conditioning	Sub-maximal (SMax) and maximal hopping	SMax Hopping—SOL+ VL Muscle Activity (% GC)		
				Short Latency—G9 vs. G12 vs. G15		
				26.89 ± 4.21 vs. 31.88 ± 4.60 vs. 33.71 ± 4.60	19%, 6%	1.13, 0.40
				Medium Latency—G9 vs. G12 vs. G15		
				21.48 ± 3.28 vs. 20.84 ± 3.37 vs. 21.37 ± 2.36	3%, 1%	0.19, 0.18
				Long Latency—G9 vs. G12 vs. G15		
				12.22 ± 3.12 vs. 10.15 ± 3.16 vs. 9.70 ± 2.94	20%, 26%	0.66, 0.15
				Maximal Hopping—SOL + VL Muscle Activity (% GC)		
				Short Latency—G9 vs. G12 vs. G15		
				18.51 ± 6.14 vs. 22.57 ± 5.81 vs. 18.63 ± 4.20	22%, 21%	0.68, 0.78
				Medium Latency—G9 vs. G12 vs. G15		
				19.12 ± 4.36 vs. 20.34 ± 3.85 vs. 20.07 ± 4.47	6%, 1%	0.30, 0.06
				Long Latency—G9 vs. G12 vs. G15		
				16.79 ± 3.47 vs. 16.59 ± 3.33 vs. 16.95 ± 4.15	1%, 2%	0.06, 0.10
Grosset et al. [93]	Co-contraction	9 sedentary adults aged 21 ± 2.3 years, 6 children aged 7 years (G7), 7 children aged 8 years (G8), 8 children aged 9 years (G9), 11 children aged 10 years (G10) and 5 children aged 11 years (G11)	MVC isometric plantar flexion	CI (TS: TA)—G7 vs. G8 vs. G9 vs. G10 vs. G11 vs. Adults		
				0.27 ± 0.03 vs. 0.26 ± 0.02 vs. 0.24 ± 0.03 vs. 0.20 ± 0.03 vs. 0.19 ± 0.04 vs. 0.13 ± 0.01 Ŧ	4%, 8%, 20%, 5%, 46%	0.40, 0.77, 1.33, 0.30, 2.45
Frost et al. [108]	Co-contraction	10 children aged 7–8 years (G1), 10 children aged 10–12 (G2), 10 children aged 15–16 years (G3)	Submaximal treadmill running	CI (running speed at 1.34 m/s)—G1 vs. G2		
				(SOL: TA) 13.5 ± 6.3 vs. 10 ± 4.7 Ŧ	35%	0.63
				(VL: H) 8.0 ± 3.2 vs. 6.5 ± 3.2 Ŧ	23%	0.47
				CI (running speed at 2.46 m/s)—G2 vs. G3		
				(SOL: TA) 16 ± 4.7 vs. 13.5 ± 7.9 Ŧ	19%	0.38
				(VL: H) 14.5 ± 7.8 vs. 8 ± 4.7 Ŧ	81%	1.01

Ŧ—Estimated from graph. Effect size (g): <0.2 (trivial), 0.20–0.59 (small), 0.60–1.19 (moderate), 1.20–1.99 (large), 2.00–3.99 (very large), and >4.0 (extremely large) [38]. (CI—co-contraction index, EMD—electromechanical delay, EMG—electromyography, GC—ground contact, GM—gastrocnemius medialis, H—hamstrings, MPF—mean power frequency, MVC—maximal voluntary contraction, SOL—soleus, TA—tibialis anterior, TS—triceps surae and VL—vastus lateralis).

**Table 4:** Effects of training on structural and neural factors in children (for multiple groups difference and effect size are expressed for consecutive pairs).

Author	Sample Age Range	Training Intervention	Findings		
			Values	Difference (%)	Effect Size (g)
Ramsay et al. [20]	CON-13, EXP-13, aged between 9–11 years	20 weeks, 3 sessions/week Circuit Training Phase 1: 70–75% 1RM Phase 2: 80–85% 1 RM Preacher curl, double leg extension, leg press, bench press, behind the neck pulldown and sit-ups/trunk curls	Muscle CSA—Baseline vs. Post-intervention (cm^2^)		
			(KE) CON: 37.5 ± 5.4 vs. 41 ± 7.2 Ŧ	9%	0.55
			EXP: 40 ± 7.2 vs. 44 ± 7.2 Ŧ	10%	0.56
			∆EXP v ∆CON		0.08
			(EF) CON: 8.6 ± 2.5 vs. 9.4 ± 1.8 Ŧ	9%	0.37
			EXP: 7.4 ± 2.9 vs. 8.2 ± 2.2 Ŧ	11%	0.31
			∆EXP v ∆CON		0.00
			MUA—Baseline vs. Post-intervention (% MUA)		
			(KE) CON: 80 vs. 79 Ŧ	1%	
			EXP: 75 vs. 86 Ŧ	15%	
			(EF) CON: 94.5 vs. 93 Ŧ	2%	
			EXP: 84 vs. 96 Ŧ	14%	
Fukunaga et al. [158]	(G1) 7 ± 0.3 years. CON-8, EXP-8 (G2) 9 ± 0.3 years. CON-8, EXP-10 (G3) 11 ± 0.2 years. CON-8, EXP-10	12 weeks, 3 sessions/week, 2/day Three maximally sustained isometric contractions of elbow flexion for 10 s	Upper Arm CSA—Baseline vs. Post-intervention (cm^2^)		
			(G1) CON: 14.4 ± 3.9 vs. 14.8 ± 4.2	3%	0.07
			EXP: 12.5 ± 2.6 vs. 13.5 ± 1.3	8%	0.28
			∆EXP v ∆CON		0.17
			(G2) CON: 16.3 ± 2.9 vs. 16.7 ± 2.7	2%	0.10
			EXP: 14.8 ± 3.0 vs. 15.9 ± 3.1	7%	0.29
			∆EXP v ∆CON		0.23
			(G3) CON: 17.6 ± 2.3 vs. 18.7 vs. 2.8	6%	0.36
			EXP: 16.6 ± 2.6 vs. 19.1 ± 3.1	15%	0.78
			∆EXP v ∆CON		0.55
Granacher et al. [159]	CON-15, aged 8.7 ± 0.5 years EXP- 17, aged 8.6 ± 0.5 years	10 weeks, 2 sessions/week, 90 min 3 sets of 10-12 reps, 70–80% 1RM Leg press, knee extension/flexion, seated calf raises, hip abduction/adduction and core exercises.	M. Quadricep CSA—Baseline vs. Post-intervention (mm^2^)		
			CON: 295.0 ± 49.7 vs. 299.4 ± 55.2	1%	0.08
			EXP: 311.0 ± 41.8 vs. 318.0 ± 14.4	2%	0.15
			∆EXP v ∆CON		0.06
Waugh et al. [157]	CON-10, aged 8.9 ± 0.3 years EXP-10, aged 8.9 ± 0.2 years	10 weeks, 2 sessions/week Plantar flexion resistance training, within a circuit, with intensity based on progressive loading starting at 8–15 RM Control group had the plantar flexion resistance training replaced by rest.	Achilles Tendon CSA—Baseline vs. Post-intervention (mm^2^)		
			CON: 40.7 ± 7.2 vs. 41.8 ± 7.9	3%	0.12
			EXP: 35.8 ± 6.3 vs. 36.7 ± 5.9	3%	0.12
			∆EXP v ∆CON		0.03
			Achilles Tendon Length—Baseline vs. Post-intervention (mm)		
			CON: 151.6 ± 32.9 vs. 153.8 ± 29.4	1%	0.05
			EXP: 160.3 ± 21.3 vs. 164.5 ± 24.3	3%	0.16
			∆EXP v ∆CON		0.07
			Achilles Tendon Stiffness—Baseline vs. Post-intervention (N/mm)		
			CON: 162.5 ± 41.8 vs. 167.4 ± 36.0	3%	0.09
			EXP: 138.4 ± 36.7 vs. 177.8 ± 31.9	28%	0.87
			∆EXP v ∆CON		0.84
McKinlay et al. [156]	CON-14, aged 12.5 ± 0.3 years EXP 1-14, aged 12.5 ± 0.7 years EXP 2-13, aged 12.6 ± 0.7 years	8 weeks, 3 sessions/week, 45 min EXP 1-Traditional resistance training, 3 sets of 8-12 reps, <80% 1RM Squats variations, lunge variations, step-ups EXP 2-plyometric training, 3 sets, 10–12 foot contacts/exercise CMJ variations, TJ variations, DJ, long jumps, jumping lunges, lateral hops	VL Thickness—Baseline vs. Post-intervention (mm)		
			CON: 20.3 ± 1.9 vs. 20.4 ± 1.7	0%	0.05
			EXP 1: 19.9 ± 2.4 vs. 21.2 ± 3.8	7%	0.47
			EXP 2: 20.1 ± 1.2 vs. 21.6 ± 3.6	7%	1.07
			∆EXP2 v ∆EXP1 v ∆CON		0.10, 0.54
			VL EMD—Baseline vs. Post-intervention (ms)		
			CON: 48.4 ± 9.5 vs. 49.7 ± 14.6	3%	0.12
			EXP 1: 47.2 ± 9.5 vs. 47.8 ± 7.0	1%	0.05
			EXP 2: 43.2 ± 7.6 vs. 40.7 ± 6.9	6%	0.28
			∆EXP2 v ∆EXP1 v ∆CON		0.35, 0.07
Ramsay et al. [20]	CON-13, EXP-13, aged between 9–11 years	20 weeks, 3 sessions/week Circuit Training Phase 1: 70–75% 1RM Phase 2: 80–85% 1 RM Preacher curl, double leg extension, leg press, bench press, behind the neck pulldown and sit-ups/trunk curls	Muscle CSA—Baseline vs. Post-intervention (cm^2^)		
			(KE) CON: 37.5 ± 5.4 vs. 41 ± 7.2 Ŧ	9%	0.55
			EXP: 40 ± 7.2 vs. 44 ± 7.2 Ŧ	10%	0.56
			∆EXP v ∆CON		0.08
			(EF) CON: 8.6 ± 2.5 vs. 9.4 ± 1.8 Ŧ	9%	0.37
			EXP: 7.4 ± 2.9 vs. 8.2 ± 2.2 Ŧ	11%	0.31
			∆EXP v ∆CON		0.00
			MUA—Baseline vs. Post-intervention (% MUA)		
			(KE) CON: 80 vs. 79 Ŧ	1%	
			EXP: 75 vs. 86 Ŧ	15%	
			(EF) CON: 94.5 vs. 93 Ŧ	2%	
			EXP: 84 vs. 96 Ŧ	14%	

Ŧ—Estimated from graph. Effect size (g): <0.2 (trivial), 0.20–0.59 (small), 0.60–1.19 (moderate), 1.20–1.99 (large), 2.00–3.99 (very large), and >4.0 (extremely large) [38]. (CMJ—countermovement jump, CSA—cross-sectional area, DJ—drop jump, EF—elbow flexors, EMD—electromechanical delay, KE—knee extensors, MUA—motor unit activation, RM—rep max, TJ—tuck jump, VL—vastus lateralis).

## Data Availability

Not applicable.

## References

[1] Binzoni T., Bianchi S., Hanquinet S., Kaelin A., Sayegh Y., Dumont M., Jéquier S. (2001). Human gastrocnemius medialis pennation angle as a function of age: From newborn to the elderly. J. Physiol. Anthr. Appl. Hum. Sci..

[2] Radnor J.M., Oliver J.L., Waugh C.M., Myer G.D., Moore I.S., Lloyd R.S. (2018). The influence of growth and maturation on stretch-shortening cycle function in youth. Sports Med..

[3] Hiort O. (2002). Androgens and puberty. Best Pract. Res. Clin. Endocrinol. Metab..

[4] Moeskops S., Oliver J.L., Read P.J., Myer G.D., Lloyd R.S. (2021). The Influence of Biological Maturity on Sprint Speed, Standing Long Jump, and Vaulting Performance in Young Female Gymnasts. Int. J. Sports Physiol. Perform..

[5] Meyers R.W., Oliver J.L., Hughes M.G., Cronin J.B., Lloyd R.S. (2015). Maximal sprint speed in boys of increasing maturity. Pediatr. Exerc. Sci..

[6] Papaiakovou G., Giannakos A., Michailidis C., Patikas D., Bassa E., Kalopisis V., Anthrakidis N., Kotzamanidis C. (2009). The effect of chronological age and gender on the development of sprint performance during childhood and puberty. J. Strength Cond. Res..

[7] Quatman C.E., Ford K.R., Myer G.D., Hewett T.E. (2006). Maturation leads to gender differences in landing force and vertical jump performance: A longitudinal study. Am. J. Sports Med..

[8] Taylor M.J., Cohen D., Voss C., Sandercock G.R. (2010). Vertical jumping and leg power normative data for English school children aged 10–15 years. J. Sports Sci..

[9] Chelly M.S., Fathloun M., Cherif N., Amar M.B., Tabka Z., Van Praagh E. (2009). Effects of a back squat training program on leg power, jump, and sprint performances in junior soccer players. J. Strength Cond. Res..

[10] Chelly M.S., Hermassi S., Shephard R.J. (2015). Effects of in-season short-term plyometric training program on sprint and jump performance of young male track athletes. J. Strength Cond. Res..

[11] Matavulj D., Kukolj M., Ugarkovic D., Tihanyi J., Jaric S. (2001). Effects of pylometric training on jumping performance in junior basketball players. J. Sports Med. Phys. Fit..

[12] Lloyd R.S., Radnor J.M., Croix M.B.D.S., Cronin J.B., Oliver J.L. (2016). Changes in sprint and jump performances after traditional, plyometric, and combined resistance training in male youth pre-and post-peak height velocity. J. Strength Cond. Res..

[13] Uzelac-Sciran T., Sarabon N., Mikulic P. (2020). Effects of 8-Week Jump Training Program on Sprint and Jump Performance and Leg Strength in Pre-and Post-Peak Height Velocity Aged Boys. J. Sports Sci. Med..

[14] Meylan C., Malatesta D. (2009). Effects of in-season plyometric training within soccer practice on explosive actions of young players. J. Strength Cond. Res..

[15] Thomas K., French D., Hayes P.R. (2009). The effect of two plyometric training techniques on muscular power and agility in youth soccer players. J. Strength Cond. Res..

[16] Daly R.M., Saxon L., Turner C.H., Robling A.G., Bass S.L. (2004). The relationship between muscle size and bone geometry during growth and in response to exercise. Bone.

[17] Dotan R., Mitchell C.J., Cohen R., Gabriel D., Klentrou P., Falk B. (2013). Explosive sport training and torque kinetics in children. Appl. Physiol. Nutr. Metab..

[18] Metaxas T.I., Mandroukas A., Vamvakoudis E., Kotoglou K., Ekblom B., Mandroukas K. (2014). Muscle fiber characteristics, satellite cells and soccer performance in young athletes. J. Sports Sci. Med..

[19] Mitchell C., Cohen R., Dotan R., Gabriel D., Klentrou P., Falk B. (2011). Rate of muscle activation in power-and endurance-trained boys. Int. J. Sports Physiol. Perform..

[20] Ramsay J.A., Blimkie C.J., Smith K., Garner S., Macdougall J.D., Sale D.G. (1990). Strength training effects in prepubescent boys. Med. Sci. Sports Exerc..

[21] Chestnut J.L., Docherty D. (1999). The effects of 4 and 10 repetition maximum weight-training protocols on neuromuscular adaptations in untrained men. J. Strength Cond. Res..

[22] Marshall P.W., McEwen M., Robbins D.W. (2011). Strength and neuromuscular adaptation following one, four, and eight sets of high intensity resistance exercise in trained males. Eur. J. Appl. Physiol..

[23] Cormie P., McGuigan M.R., Newton R.U. (2011). Developing maximal neuromuscular power: Part 1-Biological basis of maximal power production. Sports Med..

[24] Hudelmaier M., Wirth W., Himmer M., Ring-Dimitriou S., Sänger A., Eckstein F. (2010). Effect of exercise intervention on thigh muscle volume and anatomical cross-sectional areas—Quantitative assessment using MRI. Magn. Reson. Med..

[25] Jones E.J., Bishop P.A., Woods A.K., Green J.M. (2008). Cross-sectional area and muscular strength. Sports Med..

[26] Tonson A., Ratel S., Le Fur Y., Cozzone P., Bendahan D. (2008). Effect of maturation on the relationship between muscle size and force production. Med. Sci. Sports Exerc..

[27] Bojsen-Møller J., Magnusson S.P., Rasmussen L.R., Kjaer M., Aagaard P. (2005). Muscle performance during maximal isometric and dynamic contractions is influenced by the stiffness of the tendinous structures. J. Appl. Physiol..

[28] Grosset J.-F., Piscione J., Lambertz D., Pérot C. (2009). Paired changes in electromechanical delay and musculo-tendinous stiffness after endurance or plyometric training. Eur. J. Appl. Physiol..

[29] Kanehisa H., Ikegawa S., Tsunoda N., Fukunaga T. (1995). Strength and cross-sectional areas of reciprocal muscle groups in the upper arm and thigh during adolescence. Int. J. Sports Med..

[30] Kubo K., Kanehisa H., Kawakami Y., Fukanaga T. (2001). Growth changes in the elastic properties of human tendon structures. Int. J. Sports Med..

[31] Kubo K., Teshima T., Hirose N., Tsunoda N. (2014). A cross-sectional study of the plantar flexor muscle and tendon during growth. Int. J. Sports Med..

[32] Morris J. (2008). Oxford Dictionary of Sports Science and Medicine, Michael Kent. Book Rev..

[33] O’Brien T.D., Reeves N.D., Baltzopoulos V., Jones D.A., Maganaris C.N. (2010). In vivo measurements of muscle specific tension in adults and children. Exp. Physiol..

[34] Radnor J.M., Oliver J.L., Waugh C., Myer G.D., Lloyd R.S. (2021). Muscle Architecture and Maturation Influences Sprint and Jump Ability in Young Boys: A Multi-Study Approach. J. Strength Cond. Res..

[35] Kubo K., Teshima T., Ikebukuro T., Hirose N., Tsunoda N. (2014). Tendon properties and muscle architecture for knee extensors and plantar flexors in boys and men. Clin. Biomech..

[36] Radnor J.M., Oliver J.L., Waugh C.M., Myer G.D., Lloyd R.S. (2020). The Influence of Maturity Status on Muscle Architecture in School-Aged Boys. Pediatr. Exerc. Sci..

[37] Cunha G.d.S., Vaz M.A., Herzog W., Geremia J.M., Leites G.T., Reischak-Oliveira Á. (2020). Maturity status effects on torque and muscle architecture of young soccer players. J. Sports Sci..

[38] Hopkins W., Marshall S., Batterham A., Hanin J. (2009). Progressive statistics for studies in sports medicine and exercise science. Med. Sci. Sports Exerc..

[39] Krzysztofik M., Wilk M., Wojdała G., Gołaś A. (2019). Maximizing muscle hypertrophy: A systematic review of advanced resistance training techniques and methods. Int. J. Environ. Res. Public Health.

[40] Ikai M., Fukunaga T. (1970). A study on training effect on strength per unit cross-sectional area of muscle by means of ultrasonic measurement. Int. Z. Angew. Physiol..

[41] Maughan R.J., Watson J.S., Weir J. (1983). Relationships between muscle strength and muscle cross-sectional area in male sprinters and endurance runners. Eur. J. Appl. Physiol. Occup. Physiol..

[42] Wagle J.P., Carroll K.M., Cunanan A.J., Taber C.B., Wetmore A., Bingham G.E., DeWeese B.H., Sato K., Stuart C.A., Stone M.H. (2017). Comparison of the relationship between lying and standing ultrasonography measures of muscle morphology with isometric and dynamic force production capabilities. Sports.

[43] Baxter J.R., Piazza S.J. (2014). Plantar flexor moment arm and muscle volume predict torque-generating capacity in young men. J. Appl. Physiol..

[44] Fukunaga T., Miyatani M., Tachi M., Kouzaki M., Kawakami Y., Kanehisa H. (2001). Muscle volume is a major determinant of joint torque in humans. Acta Physiol. Scand..

[45] Stock M.S., Mota J.A., Hernandez J.M., Thompson B.J. (2017). Echo intensity and muscle thickness as predictors Of athleticism and isometric strength in middle-school boys. Muscle Nerve.

[46] Darby J., Li B., Costen N., Loram I., Hodson-Tole E. (2013). Estimating skeletal muscle fascicle curvature from B-mode ultrasound image sequences. Ieee Trans. Biomed. Eng..

[47] Fukutani A., Kurihara T. (2015). Comparison of the muscle fascicle length between resistance-trained and untrained individuals: Cross-sectional observation. Springerplus.

[48] Legerlotz K., Marzilger R., Bohm S., Arampatzis A. (2016). Physiological adaptations following resistance training in youth athletes—a narrative review. Pediatr. Exerc. Sci..

[49] O’Brien T.D., Reeves N.D., Baltzopoulos V., Jones D.A., Maganaris C.N. (2010). Muscle–tendon structure and dimensions in adults and children. J. Anat..

[50] Blazevich A.J. (2006). Effects of physical training and detraining, immobilisation, growth and aging on human fascicle geometry. Sports Med..

[51] Spector S.A., Gardiner P.F., Zernicke R.F., Roy R.R., Edgerton V.R. (1980). Muscle architecture and force-velocity characteristics of cat soleus and medial gastrocnemius: Implications for motor control. J. Neurophysiol..

[52] Sacks R.D., Roy R.R. (1982). Architecture of the hind limb muscles of cats: Functional significance. J. Morphol..

[53] Baroni B.M., Geremia J.M., Rodrigues R., De Azevedo Franke R., Karamanidis K., Vaz M.A. (2013). Muscle architecture adaptations to knee extensor eccentric training: Rectus femoris vs. vastus lateralis. Muscle Nerve.

[54] Guex K., Degache F., Morisod C., Sailly M., Millet G.P. (2016). Hamstring architectural and functional adaptations following long vs. short muscle length eccentric training. Front. Physiol..

[55] Abe T., Kumagai K., Brechue W.F. (2000). Fascicle length of leg muscles is greater in sprinters than distance runners. Med. Sci. Sports Exerc..

[56] Mont M.A., Cohen D.B., Campbell K.R., Gravare K., Mathur S.K. (1994). Isokinetic concentric versus eccentric training of shoulder rotators with functional evaluation of performance enhancement in elite tennis players. Am. J. Sports Med..

[57] Nasirzade A., Ehsanbakhsh A., Ilbeygi S., Sobhkhiz A., Argavani H., Aliakbari M. (2014). Relationship between sprint performance of front crawl swimming and muscle fascicle length in young swimmers. J. Sports Sci. Med..

[58] Gans C. (1982). Fiber architecture and muscle function. Exerc. Sport Sci. Rev..

[59] Kawakami Y., Ichinose Y., Kubo K., Ito M., Imai M., Fukunaga T. (2000). Architecture of contracting human muscles and its functional significance. J. Appl. Biomech..

[60] Wakeling J.M., Blake O.M., Wong I., Rana M., Lee S.S. (2011). Movement mechanics as a determinate of muscle structure, recruitment and coordination. Philos. Trans. R. Soc. B Biol. Sci..

[61] Askew G.N., Marsh R.L. (1998). Optimal shortening velocity (V/Vmax) of skeletal muscle during cyclical contractions: Length-force effects and velocity-dependent activation and deactivation. J. Exp. Biol..

[62] Earp J.E., Kraemer W.J., Cormie P., Volek J.S., Maresh C.M., Joseph M., Newton R.U. (2011). Influence of muscle–tendon unit structure on rate of force development during the squat, countermovement, and drop jumps. J. Strength Cond. Res..

[63] Earp J.E., Kraemer W.J., Newton R.U., Comstock B.A., Fragala M.S., Dunn-Lewis C., Solomon-Hill G., Penwell Z.R., Powell M.D., Volek J.S. (2010). Lower-body muscle structure and its role in jump performance during squat, countermovement, and depth drop jumps. J. Strength Cond. Res..

[64] Kawakami Y., Abe T., Fukunaga T. (1993). Muscle-fiber pennation angles are greater in hypertrophied than in normal muscles. J. Appl. Physiol..

[65] Kawakami Y., Abe T., Kuno S.-Y., Fukunaga T. (1995). Training-induced changes in muscle architecture and specific tension. Eur. J. Appl. Physiol. Occup. Physiol..

[66] Benjamin M., Kaiser E., Milz S. (2008). Structure-function relationships in tendons: A review. J. Anat..

[67] Franchi M., Trirè A., Quaranta M., Orsini E., Ottani V. (2007). Collagen structure of tendon relates to function. Sci. World J..

[68] Witvrouw E., Mahieu N., Roosen P., McNair P. (2007). The role of stretching in tendon injuries. Br. J. Sports Med..

[69] Dotan R., Mitchell C., Cohen R., Klentrou P., Gabriel D., Falk B. (2012). Child—adult differences in muscle activation—A review. Pediatr. Exerc. Sci..

[70] Couppe C., Kongsgaard M., Aagaard P., Hansen P., Bojsen-Moller J., Kjaer M., Magnusson S.P. (2008). Habitual loading results in tendon hypertrophy and increased stiffness of the human patellar tendon. J. Appl. Physiol..

[71] Roberts T.J. (2002). The integrated function of muscles and tendons during locomotion. Comp. Biochem. Physiol. Part A Mol. Integr. Physiol..

[72] Hughes D., Babbs C.F., Geddes L., Bourland J. (1979). Measurements of Young’s modulus of elasticity of the canine aorta with ultrasound. Ultrason. Imaging.

[73] Parry D., Barnes G., Craig A. (1978). A comparison of the size distribution of collagen fibrils in connective tissues as a function of age and a possible relation between fibril size distribution and mechanical properties. Proc. R. Soc. Lond. Ser. B Biol. Sci..

[74] Bailey A.J., Paul R.G., Knott L. (1998). Mechanisms of maturation and ageing of collagen. Mech. Ageing Dev..

[75] Kastelic J., Palley I., Baer E. (1980). A structural mechanical model for tendon crimping. J. Biomech..

[76] Rogol A.D., Clark P.A., Roemmich J.N. (2000). Growth and pubertal development in children and adolescents: Effects of diet and physical activity. Am. J. Clin. Nutr..

[77] Kubo K., Teshima T., Hirose N., Tsunoda N. (2014). Growth changes in morphological and mechanical properties of human patellar tendon in vivo. J. Appl. Biomech..

[78] O’Brien T.D., Reeves N.D., Baltzopoulos V., Jones D.A., Maganaris C.N. (2010). Mechanical properties of the patellar tendon in adults and children. J. Biomech..

[79] Waugh C., Blazevich A., Fath F., Korff T. (2012). Age-related changes in mechanical properties of the Achilles tendon. J. Anat..

[80] Cavanagh P.R., Komi P.V. (1979). Electromechanical delay in human skeletal muscle under concentric and eccentric contractions. Eur. J. Appl. Physiol. Occup. Physiol..

[81] Falk B., Usselman C., Dotan R., Brunton L., Klentrou P., Shaw J., Gabriel D. (2009). Child–adult differences in muscle strength and activation pattern during isometric elbow flexion and extension. Appl. Physiol. Nutr. Metab..

[82] Halin R., Germain P., Bercier S., Kapitaniak B., Buttelli O. (2003). Neuromuscular response of young boys versus men during sustained maximal contraction. Med. Sci. Sports Exerc..

[83] Waugh C.M., Korff T., Blazevich A.J. (2017). Developmental differences in dynamic muscle–tendon behaviour: Implications for movement efficiency. J. Exp. Biol..

[84] Hume P., Russell K. (2014). Overuse injuries and injury prevention strategies for youths. Strength Cond. Young Athl. Sci. Appl. Ed. Rs LloydJl Oliver. Lond..

[85] Lambertz D., Mora I., Grosset J.-F., Pérot C. (2003). An evaluation of musculo-tendinous stiffness in prepubertal children and adults, taking into account muscle activity. J. Appl. Physiol..

[86] Seger J.Y., Thorstensson A. (2000). Muscle strength and electromyogram in boys and girls followed through puberty. Eur. J. Appl. Physiol..

[87] Meyers R.W., Oliver J.L., Hughes M.G., Lloyd R.S., Cronin J.B. (2017). Influence of age, maturity, and body size on the spatiotemporal determinants of maximal sprint speed in boys. J. Strength Cond. Res..

[88] Pedley J., Lloyd R.S., Read P., Moore I., Myer G., Oliver J. (2020). A novel method to categorise stretch-shortening cycle performance across maturity in youth soccer players. J. Strength Cond. Res..

[89] Falk B., Brunton L., Dotan R., Usselman C., Klentrou P., Gabriel D. (2009). Muscle strength and contractile kinetics of isometric elbow flexion in girls and women. Pediatr. Exerc. Sci..

[90] Knight C., Kamen G. (2001). Adaptations in muscular activation of the knee extensor muscles with strength training in young and older adults. J. Electromyogr. Kinesiol..

[91] Allen G.M., McKenzie D.K., Gandevia S.C. (1998). Twitch interpolation of the elbow flexor muscles at high forces. Muscle Nerve Off. J. Am. Assoc. Electrodiagn. Med..

[92] Stokes M., Young A. (1984). The contribution of reflex inhibition to arthrogenous muscle weakness. Clin. Sci..

[93] Grosset J.F., Mora I., Lambertz D., Pérot C. (2008). Voluntary activation of the triceps surae in prepubertal children. J. Electromyogr. Kinesiol..

[94] Koh T.H., Eyre J.A. (1988). Maturation of corticospinal tracts assessed by electromagnetic stimulation of the motor cortex. Arch. Dis. Child..

[95] Henneman E. (1957). Relation between size of neurons and their susceptibility to discharge. Science.

[96] Hodson-Tole E.F., Wakeling J.M. (2009). Motor unit recruitment for dynamic tasks: Current understanding and future directions. J. Comp. Physiol. B.

[97] Milner-Brown H., Stein R., Yemm R. (1973). The orderly recruitment of human motor units during voluntary isometric contractions. J. Physiol..

[98] Grimby L., Hannerz J. (1968). Recruitment order of motor units on voluntary contraction: Changes induced by proprioceptive afferent activity. J. Neurol. Neurosurg. Psychiatry.

[99] Desmedt J.E., Godaux E. (1977). Ballistic contractions in man: Characteristic recruitment pattern of single motor units of the tibialis anterior muscle. J. Physiol..

[100] Hannerz J. (1974). Discharge properties of motor units in relation to recruitment order in voluntary contraction. Acta Physiol. Scand..

[101] Cohen R., Mitchell C., Dotan R., Gabriel D., Klentrou P., Falk B. (2010). Do neuromuscular adaptations occur in endurance-trained boys and men?. Appl. Physiol. Nutr. Metab..

[102] Bottinelli R., Pellegrino M., Canepari M., Rossi R., Reggiani C. (1999). Specific contributions of various muscle fibre types to human muscle performance: An in vitro study. J. Electromyogr. Kinesiol..

[103] Harridge S., Bottinelli R., Canepari M., Pellegrino M., Reggiani C., Esbjörnsson M., Saltin B. (1996). Whole-muscle and single-fibre contractile properties and myosin heavy chain isoforms in humans. Pflügers Arch..

[104] Kupa E.J., Roy S.H., Kandarian S.C., De Luca C.J. (1995). Effects of muscle fiber type and size on EMG median frequency and conduction velocity. J. Appl. Physiol..

[105] Lillegard W.A., Brown E.W., Wilson D.J., Henderson R., Lewis E. (1997). Efficacy of strength training in prepubescent to early postpubescent males and females: Effects of gender and maturity. Pediatr. Rehabil..

[106] Lazaridis S., Bassa E., Patikas D., Giakas G., Gollhofer A., Kotzamanidis C. (2010). Neuromuscular differences between prepubescents boys and adult men during drop jump. Eur. J. Appl. Physiol..

[107] Lloyd R.S., Oliver J.L., Hughes M.G., Williams C.A. (2012). Age-related differences in the neural regulation of stretch–shortening cycle activities in male youths during maximal and sub-maximal hopping. J. Electromyogr. Kinesiol..

[108] Frost G., Dowling J., Dyson K., Bar-Or O. (1997). Cocontraction in three age groups of children during treadmill locomotion. J. Electromyogr. Kinesiol..

[109] Fukutani A., Misaki J., Isaka T. (2016). Effect of preactivation on torque enhancement by the stretch-shortening cycle in knee extensors. PLoS ONE.

[110] Komi P.V. (2000). Stretch-shortening cycle: A powerful model to study normal and fatigued muscle. J. Biomech..

[111] Hobara H., Kimura K., Omuro K., Gomi K., Muraoka T., Iso S., Kanosue K. (2008). Determinants of difference in leg stiffness between endurance-and power-trained athletes. J. Biomech..

[112] Jones G.M., Watt D.G.D. (1971). Observations on the control of stepping and hopping movements in man. J. Physiol..

[113] Fukutani A., Kurihara T., Isaka T. (2015). Factors of force potentiation induced by stretch-shortening cycle in plantarflexors. PLoS ONE.

[114] Oliver J., Smith P.M. (2010). Neural control of leg stiffness during hopping in boys and men. J. Electromyogr. Kinesiol..

[115] Assaiante C., Amblard B. (1996). Visual factors in the child’s gait: Effects on locomotor skills. Percept. Mot. Ski..

[116] Horita T., Komi P., Nicol C., Kyröläinen H. (2002). Interaction between pre-landing activities and stiffness regulation of the knee joint musculoskeletal system in the drop jump: Implications to performance. Eur. J. Appl. Physiol..

[117] Bobbert M.F., Huijing P.A., van Ingen Schenau G. (1987). Drop jumping. I. The influence of jumping technique on the biomechanics of jumping. Med. Sci. Sports Exerc..

[118] Hreljac A. (2004). Impact and overuse injuries in runners. Med. Sci. Sports Exerc..

[119] Nicol C., Komi P., Marconnet P. (1991). Fatigue effects of marathon running on neuromuscular performance: I. Changes in muscle force and stiffness characteristics. Scand. J. Med. Sci. Sports.

[120] Hoffren M., Ishikawa M., Komi P.V. (2007). Age-related neuromuscular function during drop jumps. J. Appl. Physiol..

[121] Oliver J.L., Croix M.B.D.S., Lloyd R.S., Williams C.A. (2014). Altered neuromuscular control of leg stiffness following soccer-specific exercise. Eur. J. Appl. Physiol..

[122] Hobara H., Kanosue K., Suzuki S. (2007). Changes in muscle activity with increase in leg stiffness during hopping. Neurosci. Lett..

[123] Grosset J.-F., Mora I., Lambertz D., Pérot C. (2007). Changes in stretch reflexes and muscle stiffness with age in prepubescent children. J. Appl. Physiol..

[124] Dietz V., Noth J., Schmidtbleicher D. (1981). Interaction between pre-activity and stretch reflex in human triceps brachii during landing from forward falls. J. Physiol..

[125] McMahon J.J., Comfort P., Pearson S. (2012). Lower limb stiffness: Effect on performance and training considerations. Strength Cond. J..

[126] Henchoz Y., Malatesta D., Gremion G., Belli A. (2006). Effects of the transition time between muscle-tendon stretch and shortening on mechanical efficiency. Eur. J. Appl. Physiol..

[127] Komi P.V., Gollhofer A. (1997). Stretch reflexes can have an important role in force enhancement during SSC exercise. J. Appl. Biomech..

[128] Milner T., Cloutier C. The effect of antagonist muscle co-contraction on damping of the wrist joint during voluntary movement. Proceedings of the 17th International Conference of the Engineering in Medicine and Biology Society.

[129] Frost G., Bar-Or O., Dowling J., Dyson K. (2002). Explaining differences in the metabolic cost and efficiency of treadmill locomotion in children. J. Sports Sci..

[130] Brooks G.A., Fahey T.D., White T.P. (1996). Exercise Physiology: Human Bioenergetics and Its Applications.

[131] Ovalie W.K. (1987). The human muscle-tendon junction. Anat. Embryol..

[132] Benjamin H.J., Glow K.M. (2003). Strength training for children and adolescents: What can physicians recommend?. Physician Sportsmed..

[133] Lloyd R.S., Faigenbaum A.D., Stone M.H., Oliver J.L., Jeffreys I., Moody J.A., Brewer C., Pierce K.C., McCambridge T.M., Howard R. (2014). Position statement on youth resistance training: The 2014 International Consensus. Br. J. Sports Med..

[134] Williams T.D., Tolusso D.V., Fedewa M.V., Esco M.R. (2017). Comparison of periodized and non-periodized resistance training on maximal strength: A meta-analysis. Sports Med..

[135] Newton R.U., Kraemer W.J., Häkkinen K., Humphries B.J., Murphy A.J. (1996). Kinematics, kinetics, and muscle activation during explosive upper body movements. J. Appl. Biomech..

[136] Elliott B.C., Wilson G.J., Kerr G.K. (1989). A biomechanical analysis of the sticking region in the bench press. Med. Sci. Sports Exerc..

[137] Cormie P., McCaulley G.O., Triplett N.T., McBride J.M. (2007). Optimal loading for maximal power output during lower-body resistance exercises. Med. Sci. Sports Exerc..

[138] Campos G.E., Luecke T.J., Wendeln H.K., Toma K., Hagerman F.C., Murray T.F., Ragg K.E., Ratamess N.A., Kraemer W.J., Staron R.S. (2002). Muscular adaptations in response to three different resistance-training regimens: Specificity of repetition maximum training zones. Eur. J. Appl. Physiol..

[139] Kraemer W.J., Ratamess N.A. (2004). Fundamentals of resistance training: Progression and exercise prescription. Med. Sci. Sports Exerc..

[140] Cormie P., McGuigan M.R., Newton R.U. (2011). Developing Maximal Neuromuscular Power Part 2 Training Considerations for Improving Maximal Power Production. Sports Med..

[141] Desmedt J.E., Godaux E. (1977). Fast motor units are not preferentially activated in rapid voluntary contractions in man. Nature.

[142] McEvoy K.P., Newton R.U. (1998). Baseball throwing speed and base running speed: The effects of ballistic resistance training. J. Strength Cond. Res..

[143] Channell B.T., Barfield J. (2008). Effect of Olympic and traditional resistance training on vertical jump improvement in high school boys. J. Strength Cond. Res..

[144] Faigenbaum A.D. (2000). Strength training for children and adolescents. Clin. Sports Med..

[145] Hamill B. (1994). Relative safety of weightlifting and weight training. J. Strength Cond. Res..

[146] Lloyd R.S., Oliver J.L. (2013). Strength and Conditioning for Young Athletes: Science and Application.

[147] Palmer-Green D.S., Stokes K.A., Fuller C.W., England M., Kemp S.P., Trewartha G. (2015). Training activities and injuries in English youth academy and schools rugby union. Am. J. Sports Med..

[148] Behringer M., Heede A.v., Matthews M., Mester J. (2011). Effects of strength training on motor performance skills in children and adolescents: A meta-analysis. Pediatr. Exerc. Sci..

[149] Behringer M., vom Heede A., Yue Z., Mester J. (2010). Effects of resistance training in children and adolescents: A meta-analysis. Pediatrics.

[150] Lesinski M., Prieske O., Granacher U. (2016). Effects and dose–response relationships of resistance training on physical performance in youth athletes: A systematic review and meta-analysis. Br. J. Sports Med..

[151] Faigenbaum A.D., Lloyd R.S., MacDonald J., Myer G.D. (2016). Citius, Altius, Fortius: Beneficial effects of resistance training for young athletes: Narrative review. Br. J. Sports Med..

[152] Baker J., Cote J., Abernethy B. (2003). Sport-specific practice and the development of expert decision-making in team ball sports. J. Appl. Sport Psychol..

[153] Faigenbaum A.D., Myer G.D. (2012). Exercise deficit disorder in youth: Play now or pay later. Curr. Sports Med. Rep..

[154] Lloyd R.S., Faigenbaum A.D., Myer G., Stone M., Oliver J., Jeffreys I., Pierce K. (2012). UKSCA position statement: Youth resistance training. Prof Strength Cond.

[155] Bergeron M.F., Mountjoy M., Armstrong N., Chia M., Côté J., Emery C.A., Faigenbaum A., Hall G., Kriemler S., Léglise M. (2015). International Olympic Committee consensus statement on youth athletic development. Br. J. Sports Med..

[156] McKinlay B.J., Wallace P., Dotan R., Long D., Tokuno C., Gabriel D.A., Falk B. (2018). Effects of plyometric and resistance training on muscle strength, explosiveness, and neuromuscular function in young adolescent soccer players. J. Strength Cond. Res..

[157] Waugh C.M., Korff T., Fath F., Blazevich A.J. (2014). Effects of resistance training on tendon mechanical properties and rapid force production in prepubertal children. Am. J. Physiol. Heart Circ. Physiol..

[158] Fukunaga T., Funato K. (1992). The effects of resistance training on muscle area and strength in prepubescent age. Ann. Physiol. Anthropol..

[159] Granacher U., Goesele A., Roggo K., Wischer T., Fischer S., Zuerny C., Gollhofer A., Kriemler S. (2011). Effects and mechanisms of strength training in children. Int. J. Sports Med..

[160] Behm D.G., Faigenbaum A.D., Falk B., Klentrou P. (2008). Canadian Society for Exercise Physiology position paper: Resistance training in children and adolescents. Appl. Physiol. Nutr. Metab..

[161] Falk B., Tenenbaum G. (1996). The effectiveness of resistance training in children. Sports Med..

[162] de Salles B.F., Simao R., Miranda F., da Silva Novaes J., Lemos A., Willardson J.M. (2009). Rest interval between sets in strength training. Sports Med..

[163] Faude O., Rössler R., Petushek E.J., Roth R., Zahner L., Donath L. (2017). Neuromuscular adaptations to multimodal injury prevention programs in youth sports: A systematic review with meta-analysis of randomized controlled trials. Front. Physiol..

[164] Blazevich A.J., Wilson C.J., Alcaraz P.E., Rubio-Arias J.A. (2020). Effects of Resistance Training Movement Pattern and Velocity on Isometric Muscular Rate of Force Development: A Systematic Review with Meta-analysis and Meta-regression. Sports Med..

[165] Tillin N.A., Folland J.P. (2014). Maximal and explosive strength training elicit distinct neuromuscular adaptations, specific to the training stimulus. Eur. J. Appl. Physiol..

[166] Aagaard P., Andersen J.L., Dyhre-Poulsen P., Leffers A.M., Wagner A., Magnusson S.P., Halkjær-Kristensen J., Simonsen E.B. (2001). A mechanism for increased contractile strength of human pennate muscle in response to strength training: Changes in muscle architecture. J. Physiol..

[167] Blazevich A.J., Cannavan D., Coleman D.R., Horne S. (2007). Influence of concentric and eccentric resistance training on architectural adaptation in human quadriceps muscles. J. Appl. Physiol..

[168] Duclay J., Martin A., Duclay A., Cometti G., Pousson M. (2009). Behavior of fascicles and the myotendinous junction of human medial gastrocnemius following eccentric strength training. Muscle Nerve Off. J. Am. Assoc. Electrodiagn. Med..

[169] Seynnes O.R., de Boer M., Narici M.V. (2007). Early skeletal muscle hypertrophy and architectural changes in response to high-intensity resistance training. J. Appl. Physiol..

[170] Alegre L.M., Jiménez F., Gonzalo-Orden J.M., Martín-Acero R., Aguado X. (2006). Effects of dynamic resistance training on fascicle lengthand isometric strength. J. Sports Sci..

[171] Blazevich A.J., Gill N.D., Bronks R., Newton R.U. (2003). Training-specific muscle architecture adaptation after 5-wk training in athletes. Med. Sci. Sports Exerc..

[172] Walker S., Trezise J., Haff G.G., Newton R.U., Häkkinen K., Blazevich A.J. (2020). Increased fascicle length but not patellar tendon stiffness after accentuated eccentric-load strength training in already-trained men. Eur. J. Appl. Physiol..

[173] Proske U., Morgan D.L. (2001). Muscle damage from eccentric exercise: Mechanism, mechanical signs, adaptation and clinical applications. J. Physiol..

[174] Cormie P., McBride J.M., McCaulley G.O. (2009). Power-time, force-time, and velocity-time curve analysis of the countermovement jump: Impact of training. J. Strength Cond. Res..

[175] Kijowksi K.N., Capps C.R., Goodman C.L., Erickson T.M., Knorr D.P., Triplett N.T., Awelewa O.O., McBride J.M. (2015). Short-term resistance and plyometric training improves eccentric phase kinetics in jumping. J. Strength Cond. Res..

[176] de Villarreal E.S.S., Izquierdo M., Gonzalez-Badillo J.J. (2011). Enhancing jump performance after combined vs. maximal power, heavy-resistance, and plyometric training alone. J. Strength Cond. Res..

[177] Streckis V., Skurvydas A., Ratkevicius A. (2007). Children are more susceptible to central fatigue than adults. Muscle Nerve Off. J. Am. Assoc. Electrodiagn. Med..

